# Gene-Environment Interaction between *Arg72Pro SNP* and Selected Environmental Exposures among Brazilian Women Diagnosed with Benign Breast Disease

**DOI:** 10.31557/APJCP.2020.21.12.3477

**Published:** 2020-12

**Authors:** Rafaela Soares Senra da Costa, Rosalina Jorge Koifman, Viviane Ferreira Esteves, Marla Presa Raulino Schilling, Sergio Koifman, Ilce Ferreira da Silva

**Affiliations:** 1 *National School of Public Health Sergio Arouca, Oswaldo Cruz Foundation, Rio de Janeiro, Brazil. *; 2 *Fernandes Figueira National Institute, Oswaldo Cruz Foundation, Rio de Janeiro, Brazil. *

**Keywords:** Benign breast disease, epidemiology, public health

## Abstract

**Background:**

Benign breast disease (BBD) is a factor strongly associated with breast cancer worldwide. *Arg72Pro SNP* association with breast cancer is controversial due to the suggestion that environmental factors are required to modulate such risk. There are no studies evaluating these environmental interactions of the aforementioned SNP within BBD.

**Aim:**

To determine the frequency of *SNP Arg72Pro* in a cohort of women diagnosed with BBD; and to investigate gene-environmental interactions with environmental factors.

**Results:**

The genotype frequency was 44.6% for Arg/Pro, 39.3% for Arg/Arg genotype, and 16.3% for Pro/Pro homozygote. Gene-environment interaction analysis shows that when Arg/Arg is considered as reference, there is an ORinteraction with Arg/Pro and fabric exposure (OR=1.90;95%CI:1.04,3.48), solvents (OR=2.21;95%CI:1.01,4.83) and chlorine, bleaches, disinfectants, and liquid wax exposure (OR=2.52;95%CI:1.07,5.91). Analysis with Pro/Pro genotype as the reference showed an interaction between alcohol consumption and recessive model (OR=1.58;95%CI:1.00,2.51). Gene-environmental interactions were observed too between exposure to hair dyes, straighteners or relaxers and Arg/Arg (OR=3.26;95%CI:1.21,8.82).

**Conclusion:**

The Arg/Pro genotype was the most frequent in the BBD cohort. When compared with the Arg/Arg genotype, the presence of Arg/Pro genotype and solvents, fabric and cleaning products exposure increased the risk of BBD. When compared with Pro/Pro genotype, there were interactions between recessive model with alcohol consumption and exposure to hair products on the risk of BBD.

## Introduction

Benign breast disease (BBD) is a public health problem, since it constitutes an important breast cancer risk factor, which is the most incident neoplasm among women worldwide (Bray et al., 2018). BBD encompass heterogeneous group of lesions, which are histologically classified according to breast cancer risk (Page and Dupont, 1993). Such classification include non-proliferative lesions, proliferative lesions without atypia, and atypical hyperplasia (College of American Pathologists, 1986), representing a cancer risk varying from 0.8-1.6 among non-proliferative lesions to 2.1-25.2 among atypical hyperplasia (College of American Pathologists, 1986; Dupont and Page 1987; Palli et al., 1991; Bodian et al., 1993; Nomura et al., 1993; Minami et al., 1999; Wrensch et al., 2001). However, biological pathways leading BBD to breast cancer are still unclear.

Evidences suggest BBD occurs when biological mechanisms cannot keep cell integrity greater than 97.5% and the proliferation process remains constant. Such uncontrolled proliferation activities can originate a potentially pre-cancerous cell, determining BBD histopathological installation, or even breast cancer progression (Chapa et al., 2016). TP53 gene has a genome protective function, through protein repair or induction of apoptosis mechanisms (Donehower et al., 2019). TP53 gene is highly polymorphic, presenting 430 (Bouaoun et al., 2016) so far. There are many studies investigating cancer associations with transversion mutations of guanine (G) to cytosine (C) in exon 4 of codon 72 (rs1042522 SNP), also known as Arg72Pro SNP (Thomas et al., 1999). This SNP encodes two distinct functional alleles, arginine (Arg) and proline (Pro), which results in three distinct genotypes such as the wild genotype Arg/Arg and the mutant genotypes Pro/Pro and Arg/Pro (Thomas et al., 1999). Polymorphic variants transcribe a functionally distinct p53 protein. Evidences suggest that these distinct proteins may modulate the risk of cancer development (Dumont et al., 2003; Zhuo et al., 2009).

As far as we know, no study estimated the frequencies of TP53 SNPs in women diagnosed with BBD. However, according to the literature the frequency of TP53 polymorphisms in breast cancer range from 30–50%, with Arg72Pro SNP being the most common in this disease (Done et al., 2001; Tsuda, 2009). Moreover, it has been shown that the allele frequencies of Arg72Pro SNP vary among healthy and sick women, depending on the country and continent. Differences can be explained by the fact that these studies were developed among different ethnic populations, which may affect allele frequencies and environmental exposures (Själander et al., 1996). Authors of case-control studies developed on ethnic mixture populations, such as Brazil, observed that, when compared with women with Arg/Arg genotype, those with at least one Pro allele presented higher risk of breast cancer, ranging from 0.99 (95% CI; 0.59-1.65) in the Southeast region,(Mayorano, 2008) to 2.90 (95%CI, 1.43-3.60) in the Southern region (Damin et al., 2006). Besides the considerable ethnic variation in Brazil, environmental factors also vary throughout the country, reflecting cultural differences among Brazilian regions that influence in diet, smoking and alcohol habits, lifestyle, and environmental exposures such as those to benzene, heavy metal etc (Ellingjord-Dale et al., 2017; Kresovich et al., 2019; Ma et al., 2019). Furthermore, biological mechanisms by which this gene-environment relationship modulates the risk of breast cancer are still a target of investigation.

Despite that, no study so far evaluated the frequency of the Arg72Pro SNP in women with BBD as well as gene-environmental interactions between Arg72Pro SNP and environmental exposures among these women.Thus, the aim of our study is to determine the frequency of Arg72Pro SNP in a cohort of Brazilian women diagnosed with BBD; and to investigate gene-environmental interactions with selected environmental factors regarding the risk of benign breast disease.

## Materials and Methods

A cross-sectional study was carried out in cohorts of women diagnosed with BBD referred to the outpatient clinics of Fernandes Figueira National Institute (FFI/Fiocruz) and the Federal Hospital of Lagoa (FHL), which are BBD reference units in the city of Rio de Janeiro, Brazil. Baseline cohorts comprises women with altered breast tests (mammography, ultrasonography, and Fine Needle Aspiration – FNA) referred to FFI and FHL from July 2013 to July 2018. Histopathological confirmations were proceeded by core-biopsy, excisional biopsy, and/or surgery. The Ethics Committee Board of both FFI/Fiocruz and the National School of Public Health Sérgio Arouca/FIOCRUZ approved the protocol of this study. All included women formally agreed to participate by signing the informed consent form.

**Figure 1:**
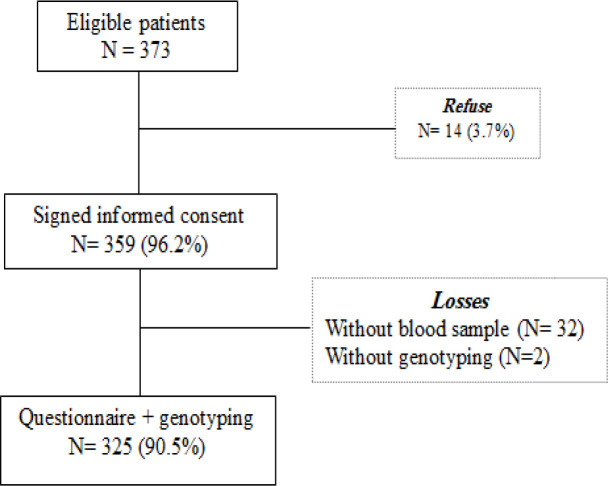
Flowchart of Patients Included in the Study

Eligibility criteria included women aged over 17 years at interview, with confirmed histopathological results of BBD. Women with history of breast cancer and/or previous BBD, and those diagnosed with cognitive conditions limiting the understanding of the informed consent form, were excluded. From 373 women included, 14 (3.7%) refused to participate in the study, whereas 359 women (96.2%) signed the informed consent form. Among the included women, 327 (91.1%) had complete questionnaire and blood sample available; however, 2 (8.9%) of them showed nonspecific bands in the amplification step and were not included in the analysis. Thus, analyses included 325 women (90.5%) who presented a complete questionnaire and DNA genotyping (Figure 1).

An interview-based questionnaire was proceeded to collect data concerning sociodemographic characteristics, clinical aspects, and selected environmental exposures (smoking habit, alcohol consumption, and domestic and occupational chemical exposures). Such instrument was adapted from validated scales (IARC Working Group on the Evaluation of Carcinogenic Risks to Humans 2004, 2010), and it was applied by three trained nurses. Clinical evaluation included weight and height measurements, using a Filizola ergometer scale, regularly calibrated according to Inmetro (1994) criteria. An inelastic tape measure assessed waist and hip measurements. Information on clinical conditions and histopathological outcomes were obtained from physical and electronic medical reports. Two 4-mL tubes of peripheral blood samples were collected, stored at 4°C, and processed at the Laboratory of Molecular Epidemiology of Cancer (ENSP/FIOCRUZ).

Genomic DNA was extracted from leukocytes using the salting out technique, and then diluted in deionized water (Miller et al., 1988). DNA quality was evaluated by spectrophotometric technique (Nanodrop®), and 0.1–10 μl of DNA was subsequently used for TP53 amplification through polymerase chain reaction (PCR) analysis. Forward and reverse primers used for amplification of the 296 bp fragment of polymorphic region were, respectively, 5’- ATCTACAGTCCCCCTTGCCG -3’ and 5’- GCAACT GACCGTGCAAGTCA-3’. Arg72Pro SNP genotyping was performed by the PCR-RFLP method (Kumar and Dunn, 1989). 

PCR was performed using approximately 1.0–4.0 μl of genomic DNA, 0.15 U of Taq-DNA Polymerase Platinum enzyme (Invitrogen, São Paulo, Brazil), 10 pmol of each primer pair, and 5 mM dNTPs in 25 μl final volume. PCR conditions were 94°C for 5 minutes for initial denaturation, followed by 35 cycles of denaturation at 94°C for 30 seconds, annealing at 68°C for 30 seconds, and extension at 72°C for 40 seconds. The final elongation step was performed at 72°C for 7 minutes. After PCR, a 4-μl aliquot was removed and digested with the BstU1 restriction enzyme (New England Biolabs, Beverly, MA) at 60°C for at least 6 hours. Digested DNA was subjected to a 3% agarose gel electrophoresis. Gels were photographed in a translucent UV light. The presence of wild Arg allele was indicated by two bands of 169 bp and 127 bp, whereas absence of digestion of mutant Pro allele was observed by a single band of 296 bp.

Sociodemographic characteristics were compared between the cohorts of women treated in IFF/Fiocruz and HFL, using the Pearson Chi-square test, and no statistically significant differences were observed. Among women included in the study, distributions of age, skin color, and BBD were compared between those whose blood samples were lost (N=34), and those who were included in the analysis (N=338), using the chi-square and Fisher’s tests; moreover, no statistically significant differences were observed.

Hardy-Weinberg Equilibrium (HWE) was estimated for Arg72Pro SNP (rs1042522), according to the reference unit, using Chi-square test (5% significance level). Statistical program R, version 3.4.3, was used in this analysis. Distributions of genotypes of the Arg72Pro SNP were at HWE in both units (Table-1). Distributions of Arg72Pro SNP genotypes were evaluated according to BBD histological type. Differences between frequencies were verified using the Chi-square test, with 5% significance level. 

Interaction odds ratios, and respective 95% confidence intervals, between Arg72Pro SNP and selected environmental factors were evaluated considering both Arg/Arg genotype and Pro/Pro genotype as reference, using case-only approach (Piegorsch et al., 1994; Yang and Khoury, 1997). Case-only design has been promoted as an efficient and valid approach to gene-environment interaction screening under the assumption of independence between exposure and genotype in the population (Piegorsch et al., 1994). If one’s primary interest is assessing possible interaction between genetic and environmental factors in the etiology of a disease, one may do so without employing control subjects (Yang and Khoury, 1997). According to Yang and Khoury (1997) the odds ratio calculated from a case-only design is related to the odds ratios for exposure alone, for genotype alone, and for their joint effects in the case-control design by the following formula:

OR_ca_ = R_ge_/(R_e_*R_g_) * OR_co_,

where OR_ca_ is the case-only odds ratio, and OR_CO_ is the odds ratio among control subjects relating exposure and the susceptibility genotype (Yang and Khoury, 1997). Assuming independence between genotype and exposure in the population, the expected value of OR_CO_ becomes unity, and the odds ratio obtained from a case-only study measures the departure from the multiplicative joint effect of genotype and exposure (Yang and Khoury, 1997). Null hypothesis considers OR_ca_ = 1; OR_ca_ > 1 if the joint effect is more than multiplicative, and ORca < 1 if the joint effect is less than multiplicative (e.g., additive) (Khoury and Flanders, 1996). Confidence intervals of case-only odds ratios can be obtained by using standard crude analyses or logistic models that control for the effects of other covariates (Yang and Khoury, 1997).

The outcome of such approach is the effect of gene-environment interaction on the risk of BBD. Thus, genetic dominance models were created to estimate the allele effect on interactions with environmental factors. When Arg/Arg genotype was considered as reference, dominant model was composed of Arg/Pro + Pro/Pro genotypes and recessive model was composed of Pro/Pro genotype. When Pro/Pro genotype was used as reference, dominant model was Arg/Arg + Arg/Pro genotypes and recessive model was composed of Arg/Arg genotype. Gene-environment interaction analyses were performed using non-conditional Logistic Regressions to estimate crude interaction odds ratio, with 95% confidence interval (95%CI). Statistical analyses were performed using Statistical Package for Social Sciences (SPSS) for Windows, version 20.0.

## Results

From 325 women included in the study, 62.8% were under 50 years old, and 37.7% self-declared reported being white. Non-proliferative lesion was the most frequent type of lesion (73.5%), followed by proliferative lesion without atypia (19.4%), and atypical hyperplasia (7.1%). The frequency of polymorphic allele (Pro) was 39.0%, whereas the frequency of Pro allele homozygous genotype was 16.7%, heterozygous 45.5%, and Arg allele homozygous genotype was 37.8% (Table 1). In both reference hospitals, genotypes distributions were in Hardy-Weinberg equilibrium (p-value>0.05).

In Table 2 we present the distribution of sociodemographic characteristics, clinical aspects, and hormonal exposures, according to the Arg72Pro SNP. In these analyses we observed that domestic and occupational exposure to hair straighteners or dyes were statistically more frequent among women with Arg/Arg genotype (96.1%) when compared with women with another genotypes (p-value=0.039).

Considering Arg/Arg genotype as reference, a strong interaction between Pro/Pro genotype and use of hormone replacement among women in menopause was observed (OR=2.16;95%CI:0.43,10.69), but without statistical significance (Table 3). Domestic and occupational exposures to solvents interacted with Arg/Pro genotype (OR=1.90;95%CI:1.04,3.48), with statistical significance (Table-3). Statically significant interaction was observed between textile mill workers or seamstresses exposure to fabric and Arg/Pro genotype (OR=2.21;95%CI:1.01,4.83). Statistically significant interactions were observed between chlorine, bleaches, disinfectants, and liquid wax exposures and Arg/Pro genotype (OR=2.52;95%CI:1.07,5.91) (Table 3).

When compared with Pro/Pro genotype, a statistically significant positive interaction was observed between current/past alcohol consumption and the recessive model (OR=1.58;95%CI:1.00,2.51) (Table 4). In addition, a strong interaction were observed between early onset of alcohol consumption (≤20 years old) and Arg/Arg genotype (OR=2.14;95%CI:0.86,5.30), but without statistical significance. Current or past smoking habit interacted with the recessive model (OR=1.60;95%CI:0.98,2.51 without statistical significance. Among those who have never smoked, a significant interaction was observed between second-hand smoking before 22 years of age and Arg/Pro genotype (OR=3.49;95%CI:0.98,12.44), without statistical significance. Hair dyes, straighteners, or relaxers exposures interacted with the recessive model (OR=3.26;95%CI:121,8.82) (Table 4).

**Table 1:** *Arg72Pro* SNP Genotype Distribution, According to Reference Units

	Observed	Expected	p-value
	N (%)	N (%)	
Overall^ a^			
Arg/Arg	127 (39.1)	123 (37.8)	0.33
Arg/Pro	145 (44.6)	154 (47.4)	
Pro/Pro	53 (16.3)	48 (14.8)	
FFI ^b^			
Arg/Arg	83 (41.3)	77 (38.3)	0.099
Arg/Pro	83 (41.3)	94 (46.8)	
Pro/Pro	35 (17.4)	30 (14.9)	
FHL ^c^			
Arg/Arg	44 (35.5)	45 (36.3)	0.709
Arg/Pro	62 (50.0)	60 (48.4)	
Pro/Pro	18 (14.5)	19 (15.3)	

^a^ Overall, Arg (p=0.61); Pro (q=0.39); b IFF, Arg (p=0.62); Pro (q=0.38); ^c^ HFL, Arg (p=0.60); Pro (q=0.39)

**Table 2:** Distribution of Sociodemographic Characteristics, Clinical Aspects, and Hormonal Exposures, According to *Arg72Pro* SNP Genotypes

Variables	Genotypes			
	*Arg/Arg* N (%)	*Arg/Pro* N (%)	*Pro/Pro* N (%)	p-value
Age				
< 50 years	78 (61.4)	91 (62.8)	35 (66.0)	0.843
≥50 years	49 (38.6)	54 (37.2)	18 (34.0)	
Self-reported skin color				
White	54 (42.5)	52 (36.1)	16 (30.2)	0.261
Nonwhite	73 (57.5)	92 (63.9)	37 (69.8)	
Histopathological				
Non-proliferative	94 (74.0)	105 (72.4)	40 (75.5)	0.872
Proliferative without atypia	24 (18.9)	28 (19.3)	11 (20.8)	
Atypic hiperplasia	9 (7.1)	12 (8.3)	2 (3.8)	
Menopause				
No	76 (60.3)	83 (58.0)	34 (64.2)	0.736
Yes	50 (39.7)	60 (42.0)	19 (35.8)	
Use of hormone replacement ^a^				
Current or past	4 (10.5)	3 (6.4)	2 (15.4)	0.57
Never	34 (89.5)	44 (93.6)	11 (84.6)	
Use of oral contraceptive				
Current or past	100 (78.7)	114 (79.2)	41 (77.4)	0.963
Never	27 (21.3)	30 (20.8)	12 (22.6)	
Alcohol consumption				
Current or past	54 (42.5)	46 (31.7)	17 (32.1)	0.146
Never	73 (57.5)	99 (68.3)	36 (67.9)	
Age of alcohol use onset ^b^				
≤20 years	32 (61.5)	28 (63.6)	7 (41.2)	0.251
>20 years	20 (38.5)	16 (36.4)	10 (58.8)	
Smoking habit				
Current or past	43 (33.9)	35 (24.1)	13 (24.5)	0.169
Never	84 (66.1)	110 (75.9)	40 (75.5)	
Smoking habit onset ^c^				
≤18 years	28 (66.7)	26 (76.5)	6 (46.2)	0.138
>18 years	14 (33.3)	8 (23.5)	7 (53.8)	
Second-hand smoking ^d^				
No	56 (66.7)	77 (70.0)	29 (72.5)	0.783
Yes	28 (33.3)	33 (30.0)	11 (27.5)	
Age of second-hand smoking onset*^ e^				
≤22 years	15 (55.6)	26 (78.8)	3 (33.3)	0.022
>22 years	12 (44.4)	7 (21.2)	6 (66.7)	
Exposure to solvents*				
No	108 (85.0)	110 (75.9)	44 (83.0)	0.143
Yes	19 (15.0)	35 (24.1)	9 (17.0)	
Exposure to hair productsf**				
No	5 (3.9%)	18 (12.7)	5 (9.4)	0.039
Yes	122 (96.1%)	124 (87.3)	48 (90.6)	
Exposure to gasoline				
No	113 (89.0)	129 (89.6)	50 (94.3)	0.524
Yes	14 (11.0)	15 (10.4)	3 (5.7)	
Exposure to fabric				
No	123 (96.9)	133 (91.7)	50 (94.3)	0.198
Yes	4 (3.1)	12 (8.3)	3 (5.7)	
Variables	Genotypes			
	*Arg/Arg* N (%)	*Arg/Pro* N (%)	*Pro/Pro* N (%)	p-value
Exposure to cleaning productsg				
No	119 (93.7)	124 (85.5)	48 (90.6)	0.086
Yes	8 (6.3)	21 (14.5)	4 (9.4)	

*, Statistically significant difference (p-value <0.05); ^a^, Only for menopausal women; ^b^, Only women who reported alcohol consumption; ^c^, Only women who reported smoking habit; ^d^, Only women who did not report smoking habit; ^e^, Only women who were exposed to second-hand smoking; and never have smoked; ^f^, Hair dyes, straighteners, or relaxers; gExposure to chlorine, bleaches, disinfectants, and liquid wax.

**Table 3:** Gene-Environment Interaction between *Arg72Pro* SNP and the Selected Environmental Factors, Considering Arg/Arg Genotype as Reference

Variables	Arg/Arg	Arg/Pro		Pro/Pro		Dominant model	Recessive model
	(N)	(N)	OR _Interaction_ (95%CI)	(N)	OR _Interaction_ (95%CI)	OR _Interaction_ (95%CI)	OR _Interaction_ (95%CI)
Use of hormone replacement ^a^							
Never	46	52	1	16	1	1	1
Current or past	4	8	1.77 (0.50,6.26)	3	2.16 (0.43,10.69)	1.86 (0.56,6.20)	1.53 (0.39,6.03)
Exposure to solvents*							
No	107	107	1	44	1	1	1
Yes	20	38	1.90 (1.04,3.48)	9	1.09 (0.46,2.59)	1.66 (0.93,2.97)	0.75 (0.35,1.64)
Exposure to fabric*							
No	117	122	1	48	1	1	1
Yes	10	23	2.21 (1.01,4.83)	5	1.22 (0.40,3.75)	1.93 (0.90,4.12)	0.75 (0.28,2.03)
Exposure to cleaning products* ^b^							
No	119	124	1	48	1	1	1
Yes	8	21	2.52 (1.07,5.91)	5	1.55 (0.48,4.97)	2.25 (0.98,5.14)	0.87 (0.32,2.37)

* Statistically significant; ^a^, Only for menopausal women; ^b^, Exposure to chlorine, bleaches, disinfectants. and liquid wax.

**Table 4:** Gene-Environment Interaction between *Arg72Pro* SNP and the Selected Environmental Factors, Considering Pro/Pro Genotype as Reference

Variables	Pro/Pro	Arg/Arg		Arg/Pro		Dominant model	Recessive model
	(N)	(N)	OR Interaction (95%CI)	(N)	OR Interaction (95%CI)	OR Interaction (95%CI)	OR Interaction (95%CI)
Alcohol consumption*							
Never	46	77	1	95	1	1	1
Current or past	17	51	1.40 (0.71,2.76)	49	1.09 (0.56,2.14)	1.23 (0.66,2.30)	1.58 (1.00,2.51)
Alcohol use onset							
Never	36	77	1	95	1	1	1
≤20 years	7	32	2.14 (0.86,5.30)	30	1.62 (0.65,4.03)	0.59 (0.34,1.02)	0.54 (0.23,1.27)
>20 years	10	18	0.84 (0.35,2.01)	18	0.68 (0.29,1.62)	0.70 (0.37,1.34)	1.33 (0.60,2.92)
Smoking habit							
Never	40	87	1	107	1	1	1
Current or past	13	41	1.45 (0.70,3.00)	37	1.06 (0.51,2.20)	1.35 (0.69,2.64)	1.60 (0.98,2.61)
Smoking habit onset							
Never	40	84	1	107	1	1	1
≤18 years	6	28	1.99 (0.76,5.22)	28	1.74 (0.67,4.53)	1.86 (0.75,4.61)	1.56 (0.88,2.77)
>18 years	7	14	0.92 (0.34,2.45)	8	0.43 (0.14,1.25)	0.65 (0.26,1.62)	1.67 (0.77,3.62)
Age of second-hand smoking onset a							
Never	29	56	1	72	1	1	1
≤22 years	3	15	2.38 (0.64,8.86)	26	3.49 (0.98,12.44)	2.98 (0.86,10.29)	0.98 (0.48,1.98)
>22 years	6	12	0.79 (0.26,2.39)	9	0.60 (0.19,1.85)	0.69 (0.25,1.88)	1.75 (0.75,4.08)
Exposure to gasoline							
No	50	116	1	126	1	1	1
Yes	3	12	1.72 (0.47,6.38)	17	2.25 (0.63,8.01)	2.00 (0.59,6.81)	1.23 (0.59,2.57)
Exposure to hair products*b							
No	5	6	1	17	1	1	1
Yes	48	122	2.12 (0.62,7.27)	124	0.76 (0.26,2.17)	1.11 (0.40,3.07)	3.26 (1.21,8.82)

* Statistically significant; ^a^, Only for women who have never smoked; ^b^, Hair dyes, straighteners, or relaxers.

## Discussion

To the best of our knowledge, this was the first study to investigate frequencies of the genotypes of Arg72Pro SNP in women diagnosed with BBD as well as gene-environmental interaction between this SNP and the selected environmental exposures. Thus, Arg/Pro genotype was the most frequent in the studied population (44.6%), followed by Arg/Arg genotype (39.1%), and Pro/Pro genotype (16.3%). Although no study evaluated the frequency of this polymorphism in women with BBD so far, Brazilian authors of case-control studies estimating *Arg72Pro* SNP frequency in women diagnosed with breast cancer observed distributions ranging from 8.0% to 55.5% for Arg/Arg genotype, from 40.3% to 60.0% for Arg/Pro genotype, and from 4.2% to 32.0% for Pro/Pro genotype (Damin et al., 2006; Mayorano, 2008; Aoki et al., 2009; Portela De Melo et al., 2009; Ramalho, 2012; Almeida et al., 2016). Brazilian breast cancer studies showed Arg/Arg genotype frequencies ranging from 44.7% to 55.5% in the South of Brazil (Damin et al., 2006; Aoki et al., 2009; Portela De Melo et al., 2009); whereas in the Southeast and Northeast regions, Arg/Pro genotype was the most frequent, varying from 41.4% to 60% (Mayorano, 2008; Ramalho, 2012; Almeida et al., 2016).

Among groups of healthy women, we observed that the Arg/Pro genotype frequency ranged from 39.2% to 58.9%. The highest frequencies were observed in Southern Brazil (46.3% to 58.9%) (Damin et al., 2006; Mayorano, 2008; Aoki et al., 2009; Portela De Melo et al., 2009; Almeida et al., 2016); whereas in the Southeastern region, frequencies of heterozygous genotype ranged from 39.2% to 42.4% (Mayorano, 2008; Almeida et al., 2016). Moreover, the Arg/Arg genotype is the second most frequent among groups of Brazilian healthy women, ranging from 33.3% to 45.0%; whereas the Pro/Pro genotype ranged from 10.3% to 16.1% (Damin et al., 2006; Mayorano, 2008; Aoki et al., 2009; Portela De Melo et al., 2009; Almeida et al., 2016). 

Differences between studies can be explained by the mixture of races in Brazil, which began with Amerindians being colonized by Portuguese peoples who brought enslaved African peoples, as well as the long history of migration of the Arab, Jewish and European peoples, and more recently, of Japanese and Chinese peoples (Layton and Smith, 2017; Braganholi et al., 2017). Such differences may be reflected in variations of genotypes frequencies observed in different regions of the country. However, different genotyping methods used among studies may also affect genotyping determination. The PCR-RFLP method is a qualitative and error-prone method in determining Arg/Pro genotype. Determination of the SNP of heterozygous genotype depends on restriction enzyme quality, which can produce a partial digestion with time. Furthermore, such method depends on the observer’s accuracy when evaluating the gel image. A replication analysis for 10% of samples was proceeded through PCR-RFLP, in order to validate the correct classification. In addition, the studied genotypes frequencies of the SNP were in HWE in the total sample as well as in each reference unit.

As breast cancer, BBD is a multifactorial disease, suggesting that a host single polymorphism might be insufficient to produce the disease phenotype, being necessary environmental factors interacting with gene polymorphism/mutations to affect the risk of disease (Ambrosone, 2007). Thus, cultural differences would play a role as in the life habits characteristic of each population, as in environmental exposures frequencies. However, biological mechanisms by which such gene-environmental interaction modulates the risk of BBD development are still unclear (Gray et al., 2017; Rodgers et al., 2018). Nevertheless, it is already known that environmental factors associated with breast cancer include endogenous and exogenous exposures to estrogen and progesterone, to tobacco and alcohol, as well as to specific chemical agents such as petroleum products, solvents, and endocrine disruptors (EDCss) found in cleaning products (Gray et al., 2017; Rodgers et al., 2018). In the present study, we observed a strong association between Arg allele and alcohol consumption, age at onset of alcohol consumption until 20 years, smoking habit, smoking onset until 18 years, passive smoking onset before 22 years, and domestic/occupational hair dye and smoothing exposures. On the other hand, the presence of at least one Pro allele was strongly associated with exposures to fabric, solvents and cleaning products such as chlorine, bleaches, disinfectants, and liquid wax. 

These findings could be partially explained by the fact that p53 protein encoded by the Arg allele is more efficient for inducing apoptosis than DNA repair (Dumont et al., 2003; Pim and Banks, 2004). Therefore, exposures to alcohol, tobacco, and second-hand smoke could interact with this allele and increase BBD risks. In addition, evidences suggest that alcohol and tobacco consumption induce DNA damage, and such consumption has been associated with increased risk of BBD and breast cancer (Pflaum et al., 2016; Ma et al., 2019). Moreover, previous studies suggested that alcohol could act in the carcinogenesis process by two pathways. Firstly by ER and PR hyperstimulation, affecting the estrogen tissue sensitivity and leading to development of ER+ breast cancer tumors, and increasing estradiol circulating levels (Ellingjord-Dale et al., 2017); secondly, via DNA damage, causing increased oxidative stress (Zhao et al., 2017). Moreover, early age at onset of alcohol consumption may reflect both a greater opportunity for prolonged exposure, and exposure during critical period of biological development in women’s breasts, in which there is greater susceptibility of BBD development (Byrne et al., 2002; Liu et al., 2012; Ellingjord-Dale et al., 2017). 

There are several evidences according to which smoking acts from initiation to neoplastic progression, mainly in cells of epithelial origin (IARC Working Group on the Evaluation of Carcinogenic Risks to Humans, 2004). However, regarding breast cancer, literature is still expanding (IARC Working Group on the Evaluation of Carcinogenic Risks to Humans, 2004; IARC Working Group, 2007). Although the specific mechanisms involved in the association between smoking and TP53 gene mutations are still unclear, the hypothesis of addition of genotoxicity associated with smoking habits seems to be plausible, since cigarettes contain about 20 carcinogens recognized by IARC, among them aromatic hydrocarbons, nitrosamines, aliphatic compounds, arylamines, and nitroarenes(IARC Working Group on the Evaluation of Carcinogenic Risks to Humans, 2004). These carcinogens act on DNA through a bond that forms adducts (Ma et al., 2019). Our results corroborate other studies whose authors reported that passive smoking was also associated with increased risk for BBD (Liu et al., 2000). Researchers have strongly suggested that the breast tissue is a target for carcinogenic effects of cigarette smoke (Conway et al., 2002), because such is more inhaled and absorbed by passive smokers, which would lead to DNA damage just as it occurs to active smokers (Johnson et al., 2011; Li et al., 2015).

Nevertheless, the p53 protein encoded by Pro allele has been mostly efficiently related to DNA repair (Siddique et al., 2005; Zhuo et al., 2009). In our study the Pro allele statistically interacted with EDCs present in a wide range of products, being found in household insecticides, pesticides, detergents, cleaning products, solvents, hair products, and plastics. EDCs are also present in occupational exposure to fabrics among textile mill workers or seamstresses, since they are used in textile manufacturing industry compounds such as textile dyes, printings, fungicides, flame retardants, solvents, plastics, and moth repellents (IARC Working Group, 1990). These compounds are capable of deregulating ER expression, PR or HER2 gene, in addition to the expression of the p53 protein. Thus, one of the hypotheses to explain such finding is that such changes may be related to BBD development, as already observed in breast cancer (Gray et al., 2017). 

Thus, according to our results, we suggest that deficiency in triggering apoptosis process in women with Arg/Pro genotype or at least one Pro allele, coupled with hormone receptors hyperstimulation promoted by solvents and cleaning products exposures could be modulating risk to BBD. Moreover, domestic or occupational exposure to hair straighteners, relaxers, or dyes interacted with the presence of at least one Arg allele (dominant model), when compared with the Pro/Pro genotype. Such finding could be explained by the fact that hair products are also considered as EDCs (Gray et al., 2017; McDonald et al., 2018). EDCs has been associated with the development of DNA adducts, which in the presence of the Arg allele, can produce p53 protein with difficulty in achieving DNA repair (Thomas et al., 1999; Dumont et al., 2003; Pim and Banks, 2004). 

Our study is the first one that described the Arg72Pro SNP distribution among women with benign breast disease, besides being pioneer in gene-environment interaction analysis between this SNP and the selected environmental exposures in BBD. For such analysis, we used the case-only approach, whose findings are an efficient and valid approach to gene-environment interaction screening under the assumption of independence between exposure and genotype in the population (Dai et al., 2018). Another advantage of this type of approach is the reduction of selection and recall bias, which are more likely to occur in case-control studies; in addition to being more efficient and less costly than a case-control study. Moreover, this approach is ideal for initial investigations of gene-environment interactions (Dai et al., 2018). Finally, another advantage of this study is the use of samples of two reference hospitals in Rio de Janeiro, with the largest sample size of Brazilian BBD studies.

However, this study has limitations that must be addressed. First, the low prevalence of hormonal replacement therapy, probably because the study population is in average very young and therefore with a low frequency of menopausal women. Another possible limitation would be the lack of statistical significance for strong gene-environment interactions between Arg72Pro SNP and important environmental factors, such as smoking habit, early age at smoking and alcohol use onset, early age at second-hand smoking, and exposure to gasoline. However, this may have occurred due to the small sample size. In addition, case only approach has as limitation that many biologically plausible modes of gene-environment interaction involve a departure from multiplicative effects and in case of additive joint effect, OR interaction derived from a case-only design can be questionable (Gauderman et al., 2019). Thus, future studies, with different study designs and larger sample size, are required to test hypotheses raised from this investigation.
